# Detecting gaps between urban expansion and lighting infrastructure growth using daytime and nighttime satellite imagery

**DOI:** 10.1016/j.jag.2026.105087

**Published:** 2026-01-06

**Authors:** Tzu-Hsin Karen Chen, Wei Chen, Eleanor C. Stokes, Yuyu Zhou

**Affiliations:** a Department of Urban Design and Planning, University of Washington, Seattle, 98105, WA, USA; b Department of Environmental and Occupational Health Sciences, University of Washington, Seattle, 98105, WA, USA; c Department of Geography, University of Hong Kong, 999077, Hong Kong, China; d NASA Headquarters, Washington, DC, 20546, USA

**Keywords:** Development disparity, Infrastructure development, Outdoor lighting, Urban expansion, Nighttime light data, Remote sensing

## Abstract

Characterizing the evolution of urban settlements is vital for informed urban planning that mitigates associated risks. Urban development has traditionally been examined in two dimensions using Earth observation: land cover change, monitored through daytime optical remote sensing, and lighting infrastructural change, observed using nighttime remote sensing. However, these two types of change have often been analyzed in isolation, limiting a comprehensive understanding of their combined impacts on urbanization. This study bridges this gap by simultaneously analyzing monthly Black Marble nighttime light (NTL) data and World Settlement Footprint data to compare lighting and urban land cover change in the Mediterranean region. Our findings reveal that 80% of urbanization-associated pixels display either urban land expansion or lighting growth, but not both. Confusion matrix highlights regional variations: commission errors are particularly high in West Asia (74%), indicating increases in nightlights driven by densification or road improvements without corresponding land conversion. Conversely, omission errors are higher in Western Europe (52%) and North Africa (47%), where urban land expansion occurs without observable lighting infrastructure growth, reflecting phenomena such as informal settlement growth, industrial infill, and energy-saving practices. This study enhances our understanding of the urbanization process through satellite observations, emphasizing the need for a more comprehensive monitoring approach that captures the diverse dimensions of urban growth.

## 1. Introduction

The Global South is projected to account for 94% of global urban population growth between 2020 and 2050 (Smit, 2021). Urbanization not only transforms land cover but also drives the expansion of modern infrastructure that is essential to residents’ well-being. For example, the global outdoor lighting market was valued at USD 17 billion in 2024 and is projected to reach USD 28 billion by 2030 (Grand View Research, 2024). The expansion of outdoor lighting has been promoted as a means to enhance safety and improve access to services (Portnov et al., 2020). However, the development of lighting infrastructure may lag behind urban expansion. In many African countries, limited access to finance and underdeveloped mortgage markets delay the installation of infrastructure, such as lighting and sewer services, years after initial housing construction (Diko and Tipple, 1992; Yeboah, 2000; Ewuoso, 2020). In informally planned areas, where housing is driven by individual or family investment rather than corporate or government-led initiatives, infrastructure provision is often uneven (Zerbo et al., 2020; Mottelson, 2023). Factors such as household financial capacity, subcontracted providers, and negotiation with authorities for public infrastructure all introduce delays and variability in infrastructure development (Yeboah, 2000).

Understanding where infrastructure lags behind urban expansion is crucial for improving resource allocation by local governments and international aid organizations, in support of the United Nations’ Sustainable Development Goal 7 (affordable energy) and 11 (sustainable cities and communities). Yet, utility infrastructure data remain scarce at scale due to fragmented supply, lack of digitization, and restricted data access (Falchetta et al., 2019). Surveys, such as those conducted by the World Bank, provide useful national estimates but are time-consuming and lack spatial granularity, limiting their usefulness in identifying localized gaps (Gao et al., 2022). These limitations underscore the urgent need for scalable and cost-effective methods to monitor infrastructure development.

Daytime satellite imagery has been widely used to map urban expansion (Pesaresi et al., 2016; Zhu et al., 2019; Chen et al., 2020; Liu et al., 2020; Reba and Seto, 2020; Güneralp et al., 2020; Marconcini et al., 2020b). Urban expansion often involves land cover change, such as the conversion of vegetated or bare soil into impervious surfaces for residential, industrial, or transportation development (Valta, 2022). Optical and radar satellite imagery can support the detection of urban land through spectral indices, classification, and texture analysis (Kaur and Pandey, 2022; Chen et al., 2023b; Gong et al., 2020; Marconcini et al., 2021). Recent integration of computer vision and remote sensing, particularly the Convolutional Neural Networks (CNNs), has significantly improved the accuracy and scalability of global urban mapping. CNN-based products, such as the World Settlement Footprint (WSF) (Marconcini et al., 2020b) and the Global Human Settlement Layer (Pesaresi et al., 2016) have been widely used to monitor urban expansion and support decisions in urban planning (García-Nieto et al., 2018), disaster management (Rentschler et al., 2023), and conservation efforts (Ren et al., 2022).

Nighttime light (NTL) data from satellites have been used to monitor economic prosperity (Elvidge et al., 2012; Mellander et al., 2015; Keola et al., 2015; Jean et al., 2016; Ji et al., 2019; Pérez-Sindín et al., 2021b; Hu and Yao, 2022), electricity access (Doll and Pachauri, 2010; Elvidge et al., 2011; Min et al., 2013; Falchetta et al., 2019; Gao et al., 2022), and infrastructure development (Bagan et al., 2019; Stokes and Seto, 2019; Zhao et al., 2020; Hu et al., 2020; Paris and Rienow, 2025; Huang et al., 2024). Unlike daytime imagery, which captures impervious surface, NTL provides insights into areas with lighting infrastructure that are observable from a nadir view, such as street lamps (Kyba et al., 2021; Levin et al., 2020). In an experimental study, street lighting was found to contribute to approximately 20% of nightlight emissions in Arizona, United States, as observed by the Visible Infrared Imaging Radiometer Suite (VIIRS) (Kyba et al., 2021). At aggregated levels, NTL brightness has been shown to correlate with electricity access rates at national, provincial, and household scales in sub-Saharan Africa (*𝑅*^2^ = 0.8) (Falchetta et al., 2019). A similar correlation was also observed in 24 villages in Henan, China (Ou et al., 2025). These findings highlight the utility of NTL as a proxy for infrastructure development. While some studies utilize NTL data to map urban extent, differences in urban extents resulting from nighttime and daytime images have been documented. For example, NTL tends to delineate the entire center of a city as urban extent due to light saturation and the lower resolution of NTL compared to daytime imagery (Zhao et al., 2020; Hu et al., 2020).

The discrepancy between daytime and nighttime observations has raised interest in identifying urban development issues. Shi et al. (2020) used discrepancies between the datasets to identify ghost neighborhoods—developed areas with low occupancy. Gao et al. (2022) combined NTL data and human settlement layers to distinguish electrified versus unelectrified regions. McCallum et al. (2022) found that 19% of the global settlement footprint lacked detectable artificial radiance in 2015, with the largest unlit shares in Africa, the Middle East, and parts of Asia. This also aligns with Blay et al. (2025)’s finding, highlighting that dark development occupies over 20% of built-up areas in some Ghanaian metropolitan cities. However, these studies rely on single-year imagery and classify areas as either lit or unlit. To date, few studies have systematically analyzed temporal change: where urban expansion occurs without concurrent lighting growth.

**Fig. 1.**
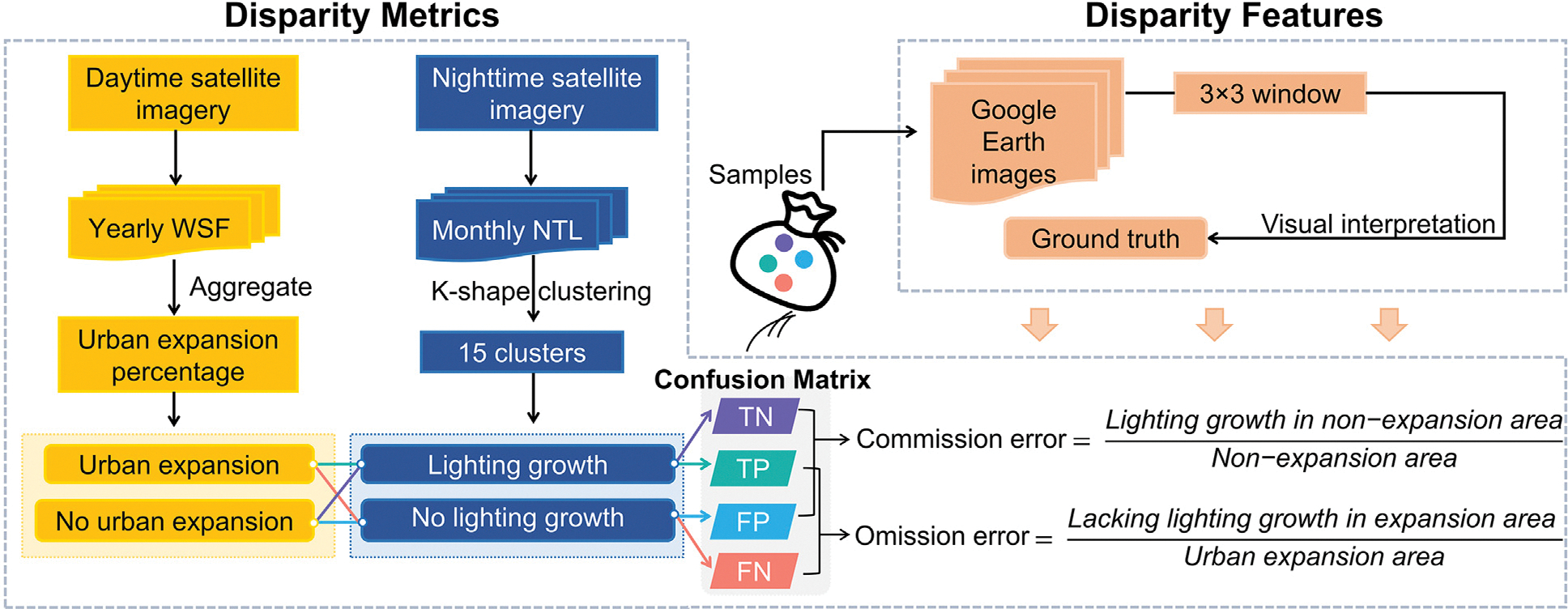
Proposed framework for detecting gaps between urban expansion and lighting infrastructure growth.

To address this gap, we propose a novel application of the confusion matrix, a tool conventionally used in classification accuracy assessment in remote sensing (Fig. 1). We simultaneously monitor two urbanization processes — urban expansion and lighting growth — using publicly available WSF data (to detect built-up areas) and monthly Black Marble NTL data (to capture lighting infrastructure). By treating urban expansion as the reference and lighting growth as the prediction, we quantify two types of mismatches: omission error, representing the percentage of newly urbanized areas lacking lighting growth; and commission error, indicating the percentage of increasingly lit areas without corresponding urban expansion. We apply this framework to the Mediterranean region to demonstrate its utility for regional development comparisons. Furthermore, we identify key features associated with the disparity in historical high-resolution imagery.

## 2. Materials and methods

### 2.1. Study areas

The Mediterranean was chosen because of its diversity of urbanization rates and forms. Urban growth is particularly acute in the south, including Algeria, Egypt, and Libya with an annual population growth rate of 3.6%. Whereas, in the northern region, annual population growth rates are 1.2%, on average, and urban development is less dense and compact (Marraccini et al., 2015). Due to the stratification of immediate and underlying factors that determine land conversion, cities in the Mediterranean region have been considered as ‘laboratories of land cover changes’ (Salvati et al., 2013).

Since 1960, the Mediterranean region, encompassing eighteen countries bordering the Mediterranean Sea, has experienced a fourfold increase in population and a twofold expansion of urban land (World Bank, 2019). As the world’s leading tourist destination and a major shipping route, the region has seen substantial infrastructure development driven by tourism and housing demand, particularly along coastal areas and near heritage cities (García-Nieto et al., 2018). Meanwhile, the Mediterranean region is warming at a higher rate, with temperatures now approximately 1.3°C above pre-industrial levels, compared to the global average of 0.85°C (Guiot and Cramer, 2016). The combined impacts of urbanization and climate change heighten the region’s vulnerability to infrastructure deficiencies.

### 2.2. Dataset

**Fig. 2.**
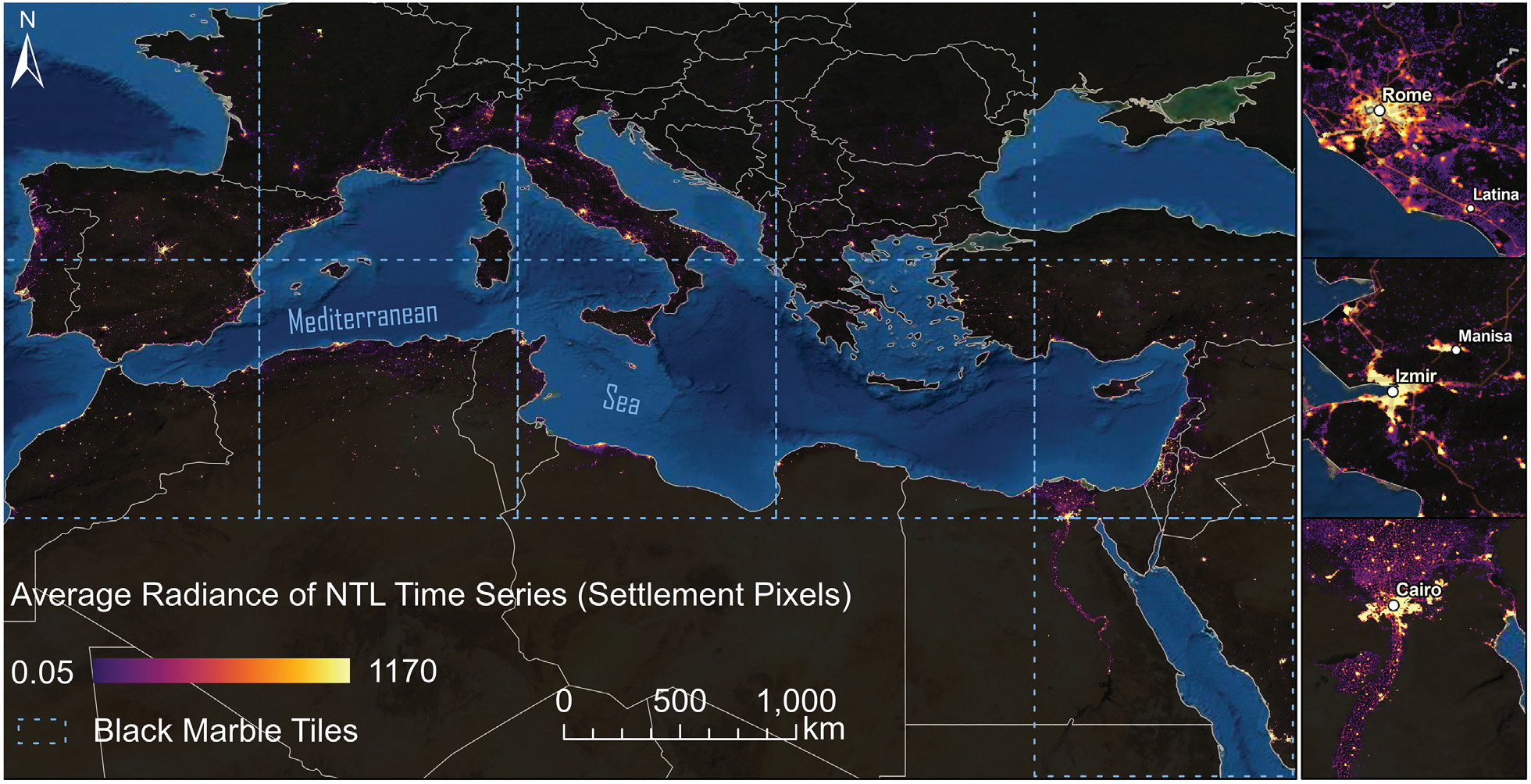
Nighttime light radiance in human settlements in the study area. The pixel value represents the average radiance of the monthly NTL images collected between January 2012 and April 2022.

To achieve full coverage of the Mediterranean coast, we selected ten NASA Black Marble VNP46A3 VIIRS tiles: h17v04, h17v05, h18v04, h18v05, h19v04, h19v05, h20v04, h20v05, h21v05, and h21v06. We collected all available 15-arc-second spatial resolution monthly DNB moonlight and atmosphere-corrected Black Marble NTL radiance (VNP46A3) at the near-nadir angle between January 2012 and April 2022 (Fig. 2). Controlling for only near-nadir view angles allows the time series signal to be more stable because recent work by the NASA Black Marble science team shows that NTL radiance can vary by 20% from view angle differences alone (Román et al., 2018). We applied three layers of quality control to ensure a reliable urban NTL time series. First, we filtered for high-quality pixel-level observations (QA flag 0 or 1) within each urban area for each month. Urban boundaries were defined by the WSF-2019 (Marconcini et al., 2020b), which delineates the extent of human settlements globally in 2019. Second, a six-month rolling window was used to smooth each urban time series and remove spurious measurements. Finally, we filtered for reliable urban NTL time series based on the number (E) and distribution (*ϵ*) of high-quality observations (Stokes and Román, 2022). E represents the proportion of high-quality observations over the entire assessment period, and *ϵ* represents the proportion of rolling 6-month windows with at least one high-quality estimate. NTL pixels in urban areas with E < 0.59 and *ϵ* < 0.93 were excluded. These thresholds were experimented and determined to balance the trade-off between keeping a high proportion of urban areas in the sample (74%) while ensuring the time series is stable, complete, and without multiple-month gaps (Stokes and Román, 2022).

To represent the land cover conversion observed from daytime satellite imagery, we collected the WSF dataset, including WSF-2015, WSF-2019, and WSF-Evolution, covering the period from 2012 to 2019. While other urban land cover datasets, such as those derived from MODIS, have a resolution more comparable to NTL data, we chose to aggregate WSF because it preserves the high-resolution, fractional estimates. We used WSF datasets to compare with the infrastructural growth identified by the NTL time series. Note that the NTL time series extends three additional years, through 2022, because we assume that electricity and lighting infrastructure are typically installed a few years after the initial site development. After an initial land cover change (such as site clearance, foundation work, and the onset of construction), it typically takes 1–3 years for the built structures to be completed and become fully functional (Tažiková et al., 2023). This time lag explains why growth in lighting signals may occur after the observed land cover change. The WSF-2015 and WSF-2019 (Marconcini et al., 2020b) were two layers outlining the extent of human settlements globally at 10-m resolution in 2015 and 2019, respectively. The WSF-Evolution (Marconcini et al., 2021) was a layer outlining the global settlement growth at 30-m resolution every year from 1985 to 2015. The accuracy and reliability of the WSF are confirmed by many international organizations, such as the World Bank, Asian Development Bank, UN-HABITAT, and International Committee of the Red Cross (Marconcini et al., 2020a).

### 2.3. Methodology

We developed a framework to assess the disparity metrics between urban expansion and infrastructural development. Subsequently, we explored the drivers of this disparity using ground truth samples extracted from Google Earth images (Fig. 1).

#### 2.3.1. Assessment of urbanization from daytime and nighttime datasets

First, we harmonized the spatial resolution of our measures for the two dimensions of urbanization: lighting infrastructure growth and urban expansion. Since NTL images have a coarser 500-m resolution, we aggregated the percentage of urban land change from 2012 to 2019 identified by the WSF to a 500-m resolution. Given the varying spatial resolutions of WSF-2015 (10-m), WSF-2019 (10-m), and WSF-Evolution (30-m), we began by extracting pixels representing percent urban area from 2012 to 2015 using the WSF-Evolution layer at 30-m resolution. These extracted pixels were then resampled to 10-m resolution using the bilinear method. Percent urban area from 2015 to 2019 was subsequently obtained by subtracting the WSF-2015 layer from the WSF-2019 layer. Finally, we combined the extracted layers from the two periods (2012–2015 and 2015–2019, both at 10-m resolution) and aggregated them to 500-m resolution to calculate the percentage of urban land change between 2012 and 2019. We masked the datasets to only include areas that have any urban land cover (Chakraborty et al., 2025).

**Fig. 3.**
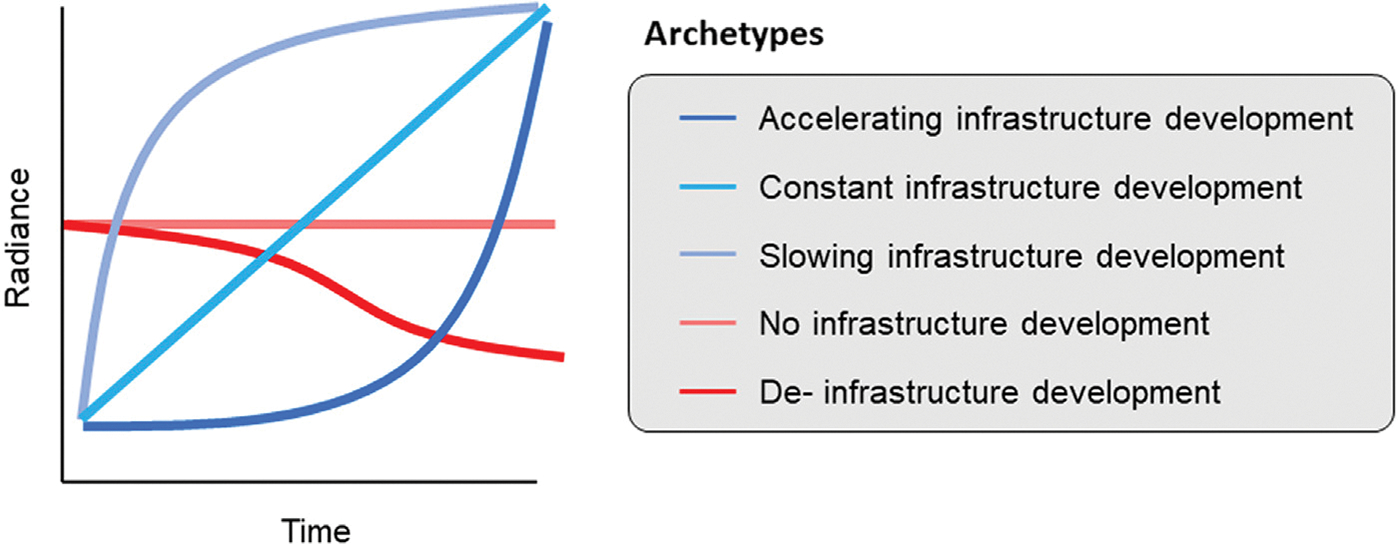
Lighting growth archetypes: Accelerating, constant, and slowing infrastructure development, and archetypes without lighting growth: No (stable) or declining infrastructure development. The clustering is based on monthly average radiance smoothed using a six-month rolling window.

Second, we employed a clustering approach on NTL time series to identify infrastructural development. We classified the resulting NTL clusters into two categories: those with lighting growth (which may include accelerating, constant, or slowing archetypes), and those without lighting growth (represented by stable or declining archetypes) (Fig. 3). These archetypes do not capture all possible forms of fluctuation. Rather, they represent a simplified set of patterns designed to distinguish the presence or absence of infrastructure change.

To classify NTL time series according to these archetypes, we utilized K-shape clustering analysis, a data-driven (i.e., unsupervised), centroid-based algorithm known for preserving the shapes of time-series sequences (Paparrizos and Gravano, 2015). This method has demonstrated comparable performance to computationally intensive hierarchical and spectral clustering techniques, which require extensive parameter tuning (Paparrizos and Gravano, 2017). We applied K-shape clustering to the NTL time series, which consisted of monthly average radiance smoothed using a six-month rolling window. Among the resulting 15 clusters (Fig. S1), clusters 0, 1, 6, 10, 12, and 13 were identified as exhibiting lighting infrastructure growth, while the remaining clusters were not.

#### 2.3.2. Disparity metrics

We proposed using a conventional remote sensing validation approach — confusion matrix — to identify coherence and disparity between lighting infrastructure growth and urban expansion (Table 1). The true positives (TP) refer to pixels that have both lighting growth based on the NTL dataset and urban expansion based on the WSF dataset. The true negatives (TN) refer to pixels that have neither lighting growth nor urban expansion. The false positives (FP) refer to pixels that do not have urban expansion but have lighting growth. The false negatives (FN) refer to pixels that have urban expansion but not lighting growth. We used these to calculate error metrics that are useful to understand regional development patterns. Omission error (Eq. (1)) answers the question: “*What percentage of urban expansion areas lack lighting growth?*” In contrast, commission error (Eq. (2)) answers “*What percentage of areas without urban expansion show lighting growth?*”. We calculated commission and omission errors by region.

(1) Omissionerror=FN∕(TP+FN)

(2) Commissionerror=FP∕(TN+FP)

**Fig. 4.**
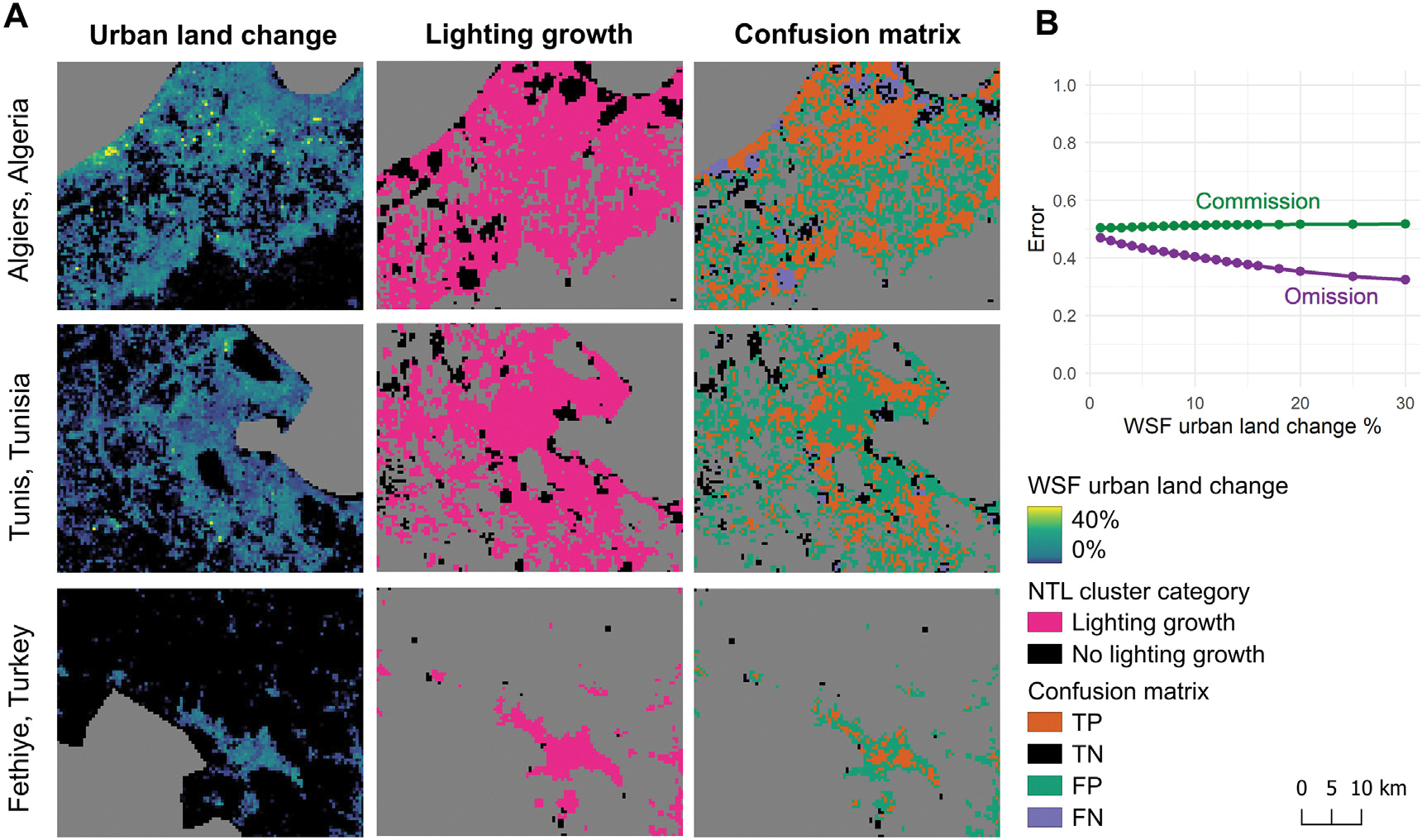
(A) Comparison of urbanization detected from daytime imagery (urban land change) and nighttime imagery (lighting growth), along with the corresponding confusion matrix: TP (urban expansion with lighting growth), TN (no urban expansion or lighting growth), FP (lighting growth without urban expansion), and FN (urban expansion without lighting growth). (B) Classification errors across different percentages of urban land change.

These metrics are based on binary classification: the presence or absence of lighting growth and the presence or absence of urban expansion. To achieve this, we classified the NTL clusters into binary categories: with lighting growth (accelerating, constant, or slowing) and without lighting growth (stable or declining). For binary urban expansion categories, we searched the threshold for the percentage of urban land change, ranging from 0 to 100% within 500-m resolution pixels. To evaluate the impact of thresholds on the disparity metrics, we incrementally increased the threshold from 3% to 100%. Initial experiments revealed that omission errors saturated at approximately 20% (Fig. 4), indicating that lighting growth is typically observable when the percentage of urban land change exceeds this threshold. Therefore, we adopted a 20% threshold for ground-truthing analysis to examine the drivers of disparity based on high-resolution Google Earth images. For summarizing regional statistics, we adopted the 5% threshold using the elbow method (Kodinariya et al., 2013), which balances the commission and omission errors (Fig. 4).

#### 2.3.3. Ground-truthing NTL change

**Fig. 5.**
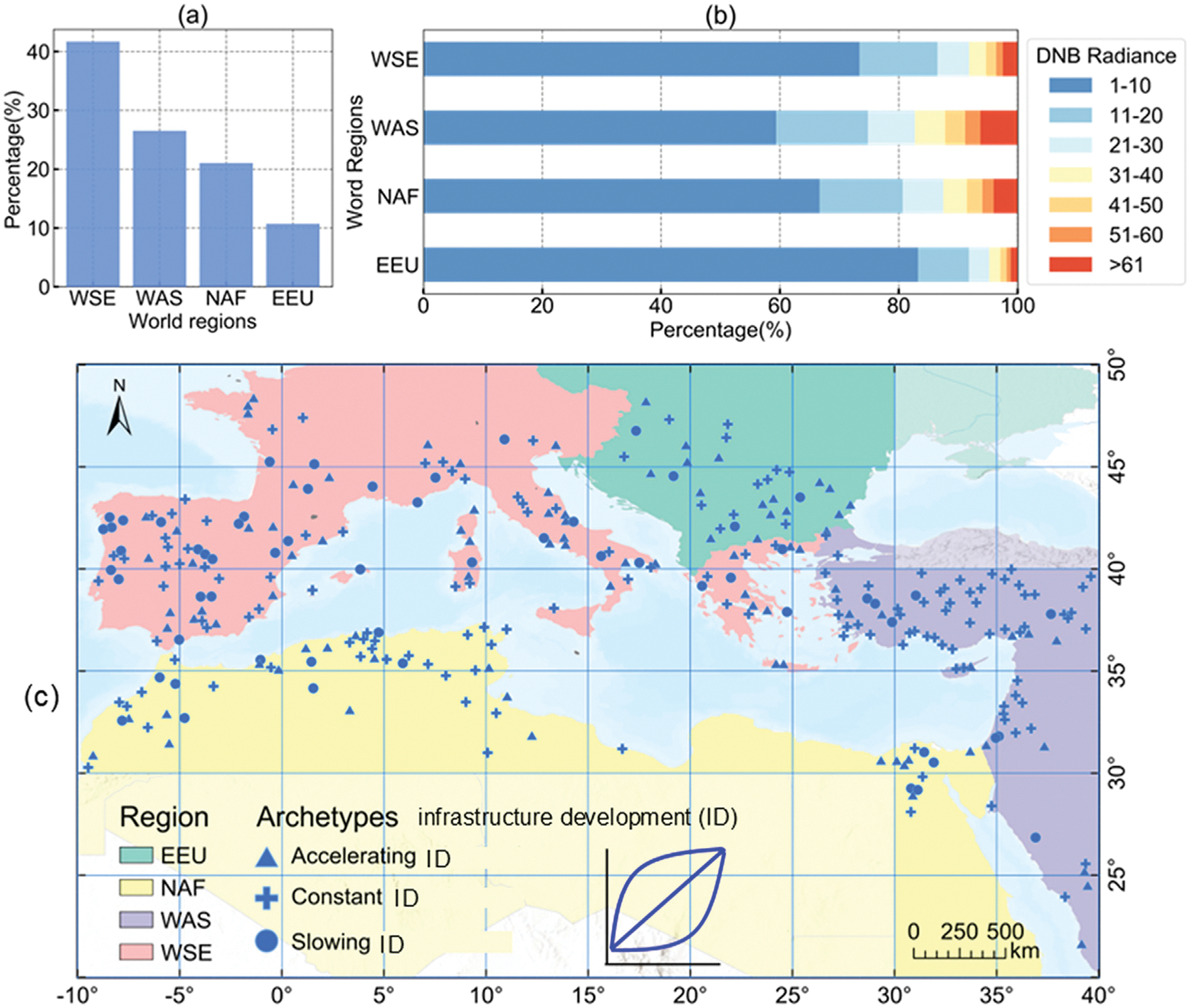
(a) The percentage of NTL-urbanization pixels by regions; (b) The percentage of NTL-urbanization pixels at 10-unit intervals of digit number; (c) Spatial distribution of 300 samples.

To validate the infrastructural development identified from NTL time series, we manually identify any urban land or lighting infrastructure change on Google Earth images, which was used as ground truth. We collected a total of 300 samples without replacement from pixels classified under accelerating, constant, and slowing lighting growth clusters (Fig. 5c). We utilized Google Earth images within a 3 × 3 window from 2012 to 2022 to interpret their urban expansion status. The sampling strategy we adopted was based on the distribution of all available non-zero NTL pixels in the Mediterranean coast, which exhibited geographically uneven (Fig. 5a). Furthermore, the numbers of non-zero NTL pixels at 10-unit intervals of digital number varied across different regions (Fig. 5b). To address these factors, we implemented a stratified random sampling scheme for selecting sampling points. This involved stratifying the samples first by region and then by intervals of NTL data values during the study period.

#### 2.3.4. Analysis of disparity features

**Fig. 6.**
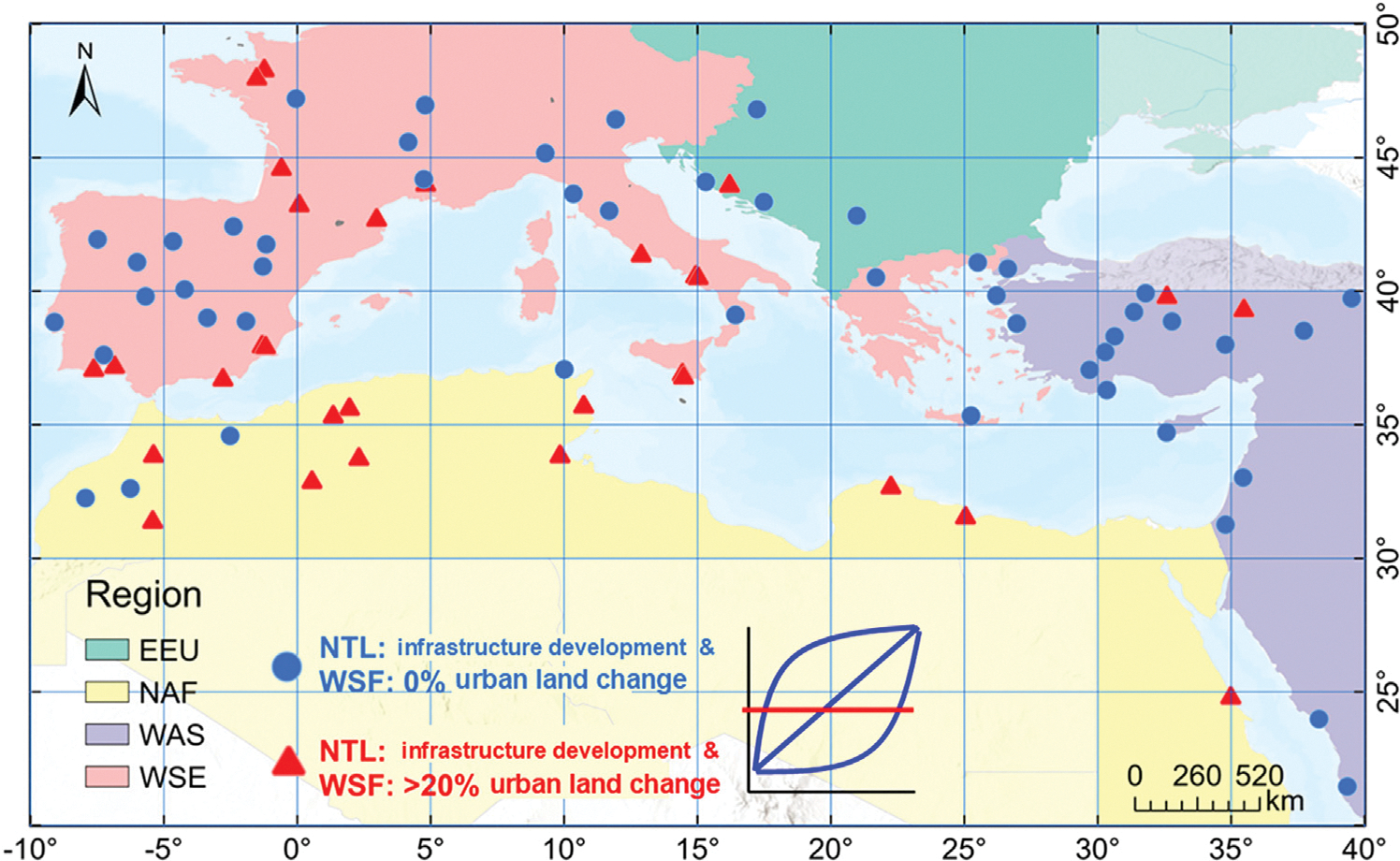
Spatial distribution of samples used for comparison including 50 samples for lighting growth without urban expansion and 30 samples for urban expansion without lighting growth.

To investigate the disparities between infrastructural development identified from NTL time series and urban expansion identified from the WSF dataset, we collected a total of 80 samples, which yield a standard error of approximately 5.5%, calculated following the sample size equation in Olofsson et al. (2014). The number of samples was determined as a balance among representativeness, available labor resources, and the desired precision of the results. First, we collected 50 samples using the aforementioned sampling strategy from pixels classified as lighting growth from NTL data but with 0% percentage of urban land cover change based on the WSF (Fig. 6). Additionally, we collected 30 samples using the same sampling strategy from pixels classified as no lighting growth from NTL data but had a percentage of urban land change greater than 20%. The difference in sample size between the two groups was chosen based on their varying dominance. To check the realistic status of urban expansion, we used Google Earth Images as a reliable reference.

## 3. Results

### 3.1. Different urbanization patterns identified by nighttime and daytime images

The confusion matrix reveals that 80% of the urbanizing areas do not exhibit both urban expansion and lighting growth concurrently. The commission error exceeds half (50%–52%) (Fig. 4), indicating that most areas showing lighting growth are within existing urban areas. The threshold used to define urban expansion has only a minor effect on the commission error, which ranges from 50% at 1% land cover change threshold to 51% at thresholds of 8% or higher. In contrast, omission errors range from 32% to 47%, suggesting that in areas with increased impervious surface, more than half also experienced a corresponding increase in NTL. Although omission errors are relatively lower than commission errors, they raise greater concerns for residents’ well-being (particularly in low- and middle-income countries), as they may indicate inadequate development of electricity infrastructure in new urban areas. Validation metrics highlight regional variations (Table 2). Commission errors are particularly high in West Asia (74%), reflecting increases in nightlights driven by densification or road improvements without corresponding land cover change (Fig. 8). Conversely, omission errors are higher in Western Europe (52%) and North Africa (47%), where urban land expansion occurs without increases in lighting infrastructure, reflecting phenomena such as insufficient infrastructure, non-residential infill, and energy-saving practices (Fig. 10).

### 3.2. Ground-truthing of urbanization with lighting growth

**Fig. 7.**
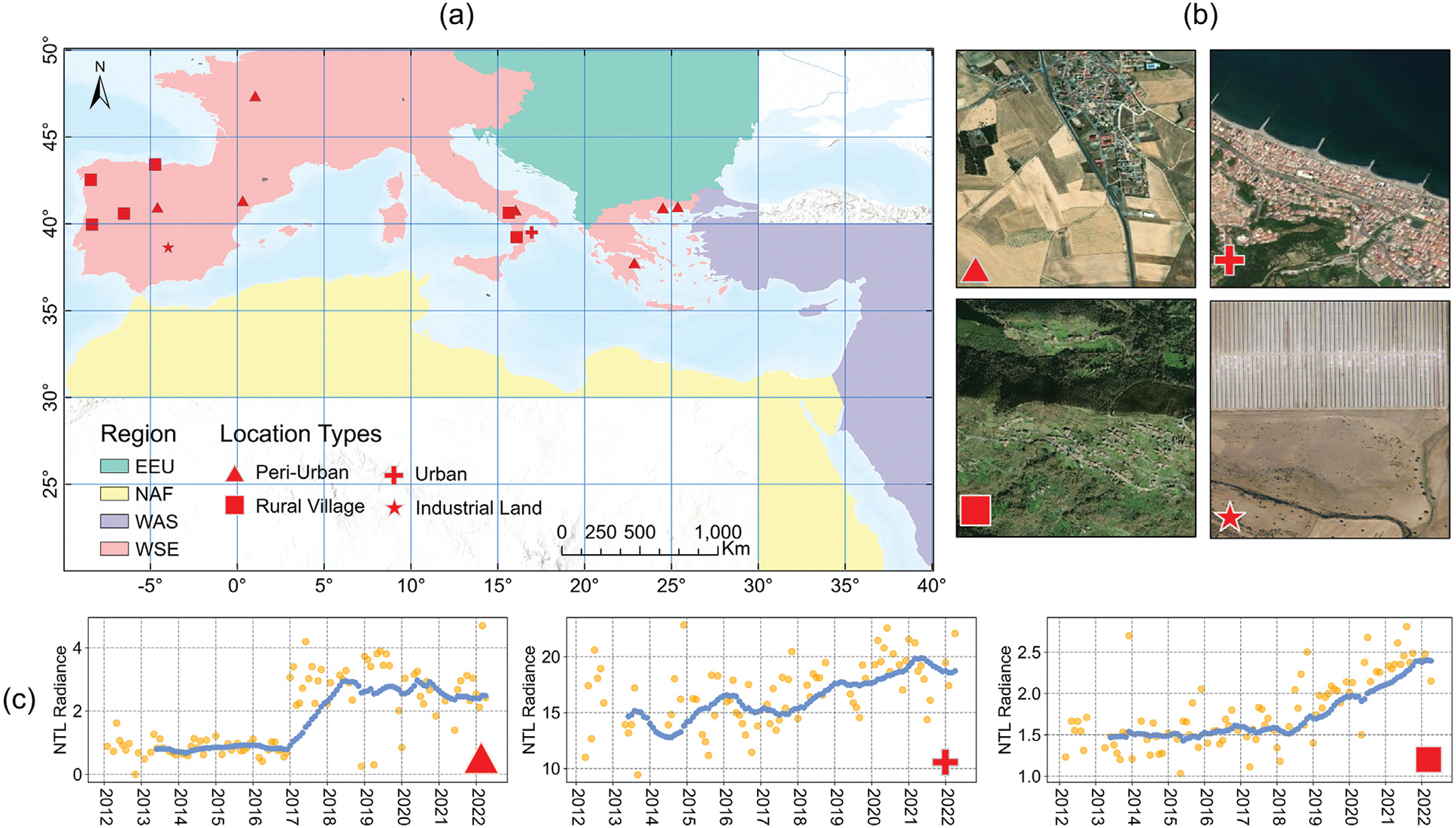
(a) Spatial distribution of ground-truthing samples without urbanization-related signatures in Google Earth images; (b) Google Earth images of samples located in four different location types and (c) examples of NTL time series of three location types. Yellow dots indicate raw observations, and blue dots represent data after smoothing.

When the NTL time series indicated lighting growth for a pixel, our remote sensing expert has a 95% probability of identifying signs of land cover or infrastructure-related changes in Google Earth time series images (Fig. 7a). Although an increase in NTL is a direct proxy of lighting infrastructure growth, it also correlates with the emergence of new built-up areas, as shown by the overlaps between daytime (WSF) and nighttime (NTL) urbanization results. In Google Earth images, visible changes associated with lighting growth included initial land conversion, land use intensification, redevelopment, road improvement, and construction of public infrastructure. However, we found that a small proportion (5%) of the samples with increased NTL did not have observable urbanization in Google Earth images. One possible explanation is that some NTL changes are spectral or related to the design or abundance of exterior lighting, which may not be detectable through daytime Google Earth images. For example, revitalization efforts, such as increasing the luminosity of street lighting, were distributed in rural villages in European countries (Fig. 7b and c). Transportation corridor lighting development can also cause increased NTL levels but with no discernible changes in Google Earth images.

### 3.3. Lighting growth without urban land expansion

**Fig. 8.**
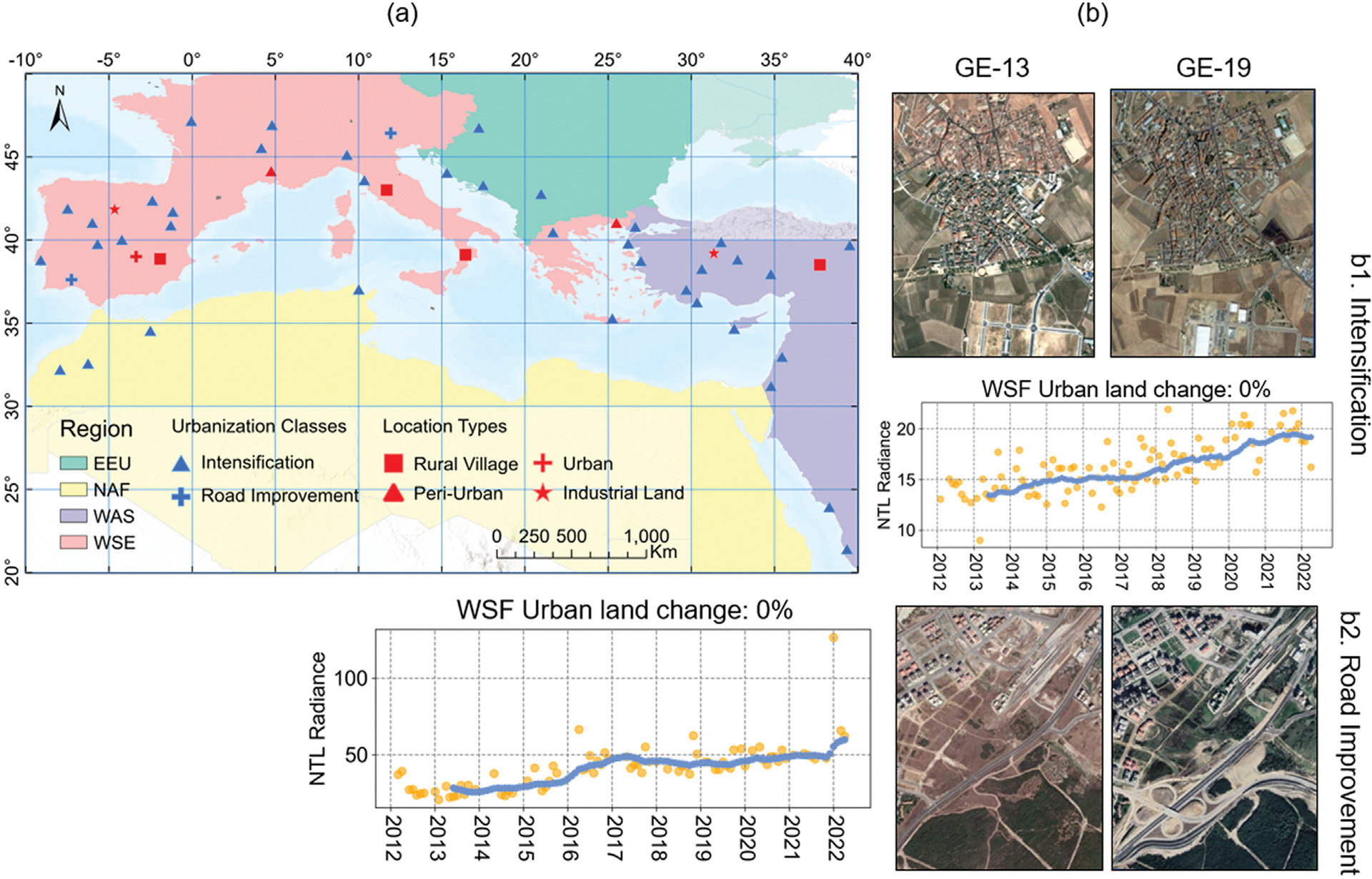
(a) Spatial distribution of samples where nighttime images show urbanization-related signatures but daytime images suggest an absence of urbanization; (b1) Examples of intensification; (b2) road infrastructure investment were identified by nighttime images but not by daytime images. Yellow dots indicate raw observations, and blue dots represent data after smoothing.

NTL time series detect urbanization resulting from densification and road improvement, leading to 40% more pixels showing urbanization patterns in nighttime images but not in daytime images (Fig. 8a). Regionally, lighting growth without urban land expansion is most common in West Asia, accounting for 58% of the region’s total land area (Table 3). Economic development and population densification typically occur after the initial land conversion. Thus, densification, characterized by population and electricity usage increases, might explain the lighting growth without substantial urban land expansion. In some cases, building density increases in an already dense built environment might not reflect in WSF due to resolution constraints. As Fig. 8b1 shows, building intensification occurred between 2013 and 2019, but the percentage of urban land change in the WSF during this period was 0. In addition, improving roads can lead to the growth of lighting infrastructure without land conversion. Fig. 8b2 illustrates the construction of an elevated road between 2013 and 2019, resulting in an increased NTL level, while the WSF observed no urban land change. Most of these cases were found in rural villages and peri-urban areas.

### 3.4. Urban land expansion without lighting growth

**Fig. 9.**
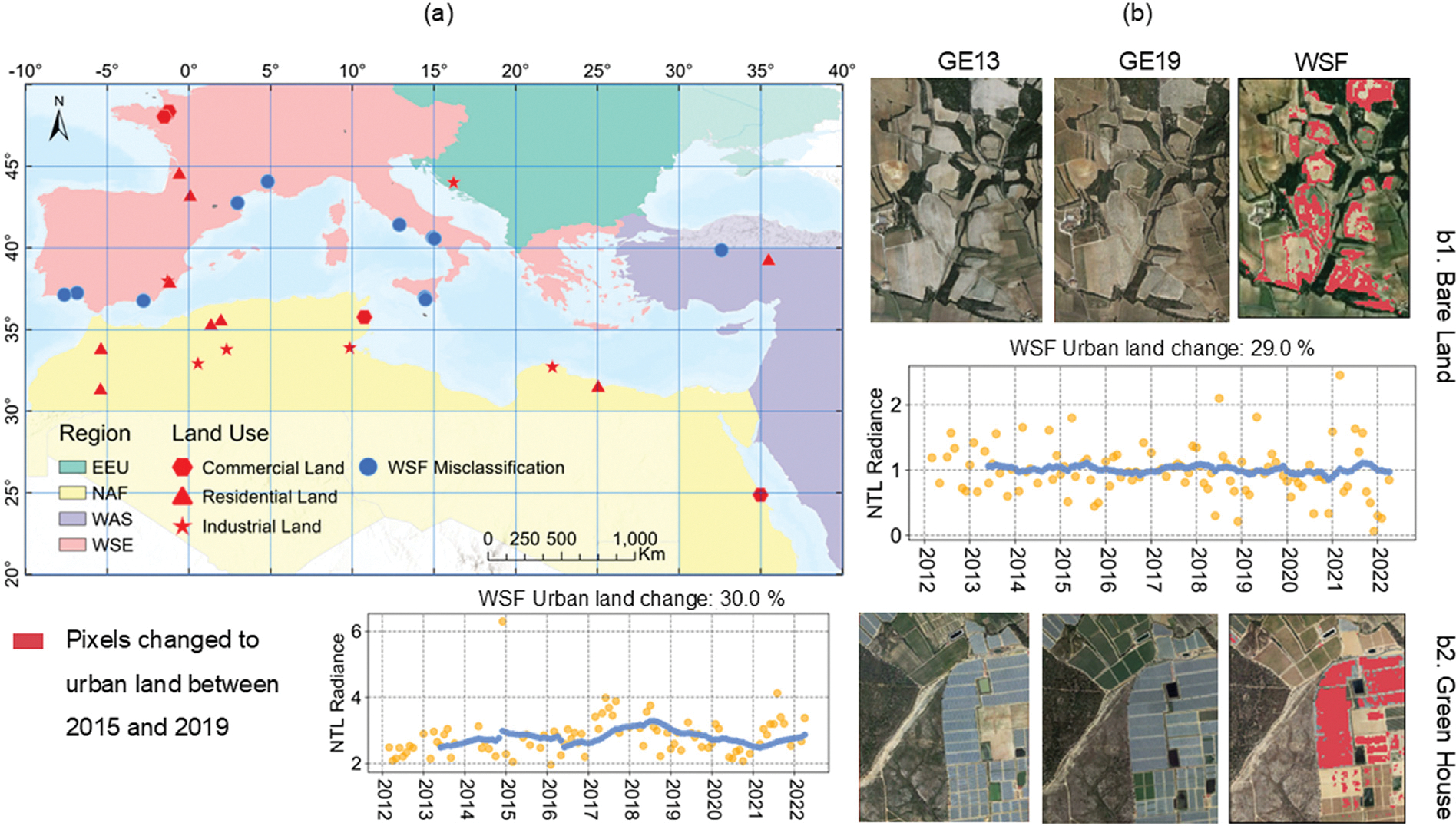
(a) Spatial distribution of samples where daytime images show urban land change higher than 20%, but nighttime images suggest an absence of urbanization; (b) Examples of misclassification of initial land change from the WSF layer. Yellow dots indicate raw observations, and blue dots represent data after smoothing.

**Fig. 10.**
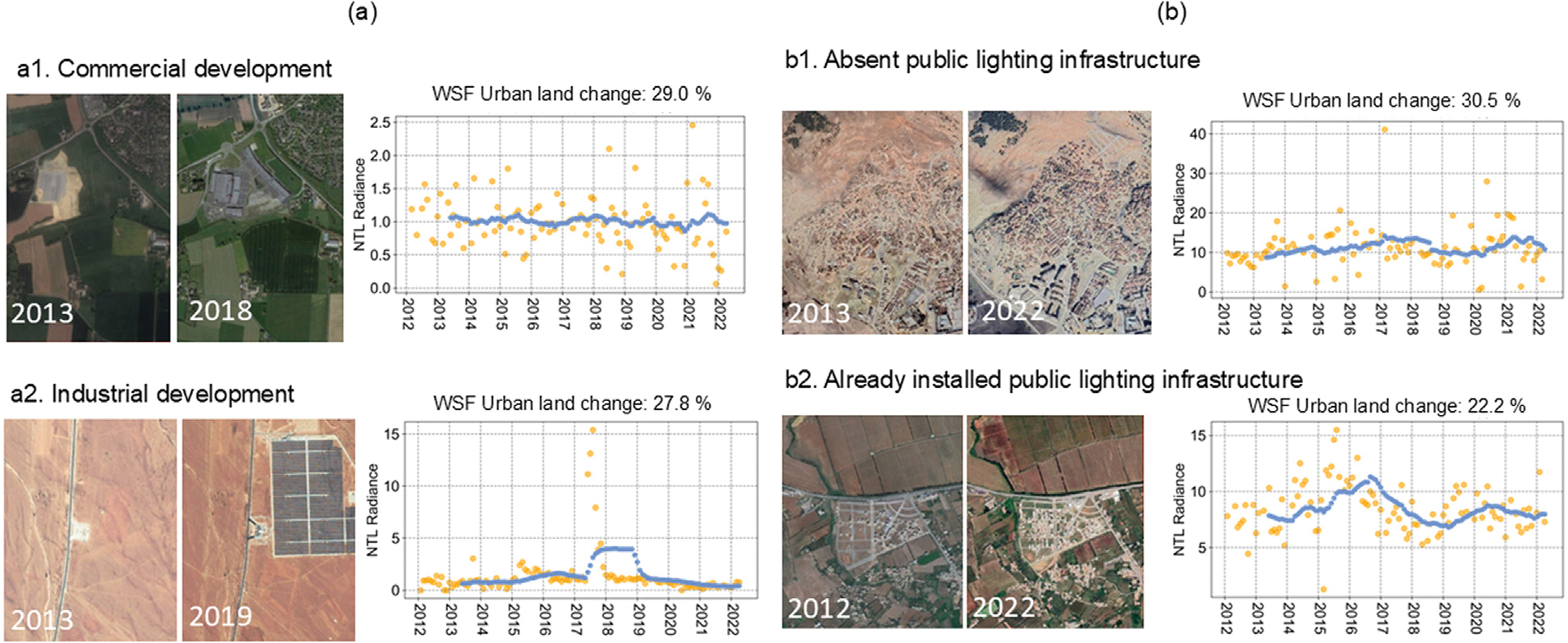
Examples of (a1) new commercial, (a2) industrial, (b1)informal settlement, and (b2) formal residential development without increases in NTL time series signals. Yellow dots indicate raw observations, and blue dots represent data after smoothing.

Urban land expansion without lighting growth is particularly prevalent in North Africa, where it accounts for 17% of the region’s total land area (Table 3). The omission error — the percentage of new urban areas identified through WSF lacking lighting growth as indicated by NTL — can be attributed to industrial development, dark growth, and misclassification within the WSF. Misclassification in the WSF-2019 product accounted for 33% of the samples exhibiting urban land change greater than 20% without corresponding increases in NTL levels (Fig. 9). These misclassified samples were primarily located in Europe, where greenhouses and bare land were incorrectly categorized as urban areas in the WSF-2019 product. The remaining 66% of samples showing urban land change without NTL increases can be explained by commercial or industrial development and dark growth. Some commercial or industrial developments involved the construction of a single large building, which did not increase observable NTL levels (Fig. 10a1,a2). Fig. 10b1 illustrates informal settlement growth without public lighting infrastructure, a phenomenon commonly observed in small towns in African cities where energy availability is limited. Dark growth can also occur in suburban residential neighborhoods in parts of European cities, where streetlights are not extensively installed. This may reflect energy-saving practices or cultural preferences for lower light usage (Fig. 10b2).

## 4. Discussion

This study presents using daytime and nighttime satellite imagery. Our findings reveal that such disparities are common rather than exceptional: 80% of the urbanization-associated pixels present either urban expansion or lighting growth, but not concurrently over the Mediterranean region (Table 3). In the past two decades, both daytime and nighttime satellite data have been extensively applied to study urbanization. However, most studies have relied on either one or the other (Zhang and Seto, 2013; Chen et al., 2023c), and efforts to integrate both have typically focused on improving classification accuracy (Zhang et al., 2015; Zheng et al., 2021; Huang et al., 2021). By contrast, our study contributes to the understanding of urbanization as a multi-dimensional process (Stokes and Seto, 2019). We highlight the complementary nature of daytime and nighttime data and demonstrate that their integration — via a confusion matrix approach — can reveal mismatches between urban expansion and lighting growth. This technique enables scalable, transboundary analysis that may support resource allocation and infrastructure development, especially in regions where administrative data are scarce or incomplete.

Our finding shows that more than 50% of existing urban areas show continued lighting growth. This aligns with prior studies suggesting that NTL tends to detect larger urban footprints than daytime imagery by 30%–760% in some cases due to its saturation and lower spatial resolution, especially before the advent of VIIRS in 2012 (Hu et al., 2020; Uchiyama and Mori, 2017). Overestimated footprint through NTL may cluster in larger cities (Bagan et al., 2019; Uchiyama and Mori, 2017; Zou et al., 2017), city centers (Zhao et al., 2020), and suburbs (Bagan et al., 2019). However, we argue that the phenomenon cannot be explained by resolution and light saturation alone. Nightlight captures different facets of urban dynamics, such as economic development (Pérez-Sindín et al., 2021a), industrial land use (Paris and Rienow, 2025), and population densification (Stokes and Seto, 2019), which do not always correspond with surface imperviousness. Long-term lighting increases without changes in built-up area may signal broader socio-economic transformations that daytime images alone cannot reveal.

This result might also be biased due to the underestimation of urban expansion in the WSF dataset. While WSF 2015 reports overall accuracies exceeding 85% (Marconcini et al., 2020b), region-specific assessments have identified low detection rates for refugee settlements (10%) and small settlements in mountainous regions (47%) (Van Den Hoek and Friedrich, 2021; Chen et al., 2023a). Similar challenges have been documented for WSF-Evolution, which demonstrates reduced temporal accuracy in monitoring small settlements over time (R^2^ = 0.45) (Chen et al., 2023a). These limitations suggest two potential sources of false alarms in our analysis: (1) Non-urban expansion with lighting growth, which may be overestimated when small-scale settlement expansion is missed; and (2) No urban expansion or lighting growth, which may be overestimated given NTL’s low sensitivity to dimmer lights commonly found in rural areas. With rural and small-scale settlements home to approximately 3.4 billion people — many of whom are disproportionately affected by infrastructure deficits — future work should aim to improve methods that better capture such contexts (UN, 2019).

Conversely, our results also show 40% of urban expansion occurred without lighting growth. This pattern is pronounced in North Africa, where omission errors exceed 47% and found common in small towns. Energy provision and maintenance remain persistent challenges in the region, as governments struggle to meet the demands of rapid development (El-Bouayady and Radoine, 2023). Our results complements previous findings of “dark development” in Ghanaian Inland cities (Blay et al., 2025). One caution we note is that a lack of lighting growth may not be necessarily indicate a lack of infrastructure. Some studies have found low NTL in rural areas can result from limited economic activity (Paris and Rienow, 2025) or housing vacancy (Shi et al., 2020), even where electricity infrastructure exists.

Notably, we also observe a high omission error in parts of Western Europe, especially in suburban areas. This may reflect cultural preferences for lower outdoor lighting or energy conservation policies (Mills and Schleich, 2012). The European Union’s Energy Efficiency Directive made its targets legally binding, requiring a reduction in energy consumption of at least 20% by 2020 compared to 2014 levels, and an additional 11.7% reduction by 2030 relative to the 2020 baseline (Tsemekidi Tzeiranaki et al., 2025). Improvements in LED lighting technology and magnitude reduction have been key contributors to these goals—for example, lighting accounts for approximately 20.3% of the total decrease in electricity consumption in Spain between 2009 and 2016 (Moral-Carcedo and Pérez-García, 2021; Montoya et al., 2017). Our findings support previous work showing dimmer lighting in Western European suburbs despite high residential density (Zhao et al., 2020), and reinforce that NTL intensity can reflect not only urban form but also behavioral and regulatory factor (Zhang et al., 2022). This contradicts earlier global studies suggesting that higher-density environments tend to show stronger agreement between daytime and nighttime urban extent (Bagan et al., 2019). We suggest that such relationships are not universal and should be examined within regional context. Although the specific drivers may differ by context, our framework provides a scalable first-pass tool to identify regions where infrastructure development potentially lags behind land development.

Despite its contributions, this study is not without limitations. First, the drivers of disparities between urban expansion and lighting growth (e.g., informal settlement development, urban densification, and road improvements) cannot be fully explained without high-resolution imagery, qualitative data, and local knowledge (Paris and Rienow, 2025).

Second, increases in NTL levels are not solely attributable to lighting infrastructure. Non-electric and transient light sources such as vehicles, fires, gas flares, and fishing vessels (Bhattarai et al., 2023), as well as natural phenomena including volcanic eruptions, lightning, and moonlight (Levin et al., 2020). Sensor limitations further complicate interpretation because satellite sensors primarily detect upward-directed radiation, making external lighting more detectable than illumination shielded by building interiors (Kuechly et al., 2012). Additional sources of uncertainty — such as atmospheric interference, seasonal variability, spectral shifts from LED adoption, and mismatches between satellite overpass time and local lighting peaks — also affect temporal consistency (Cao and Bai, 2014; Levin et al., 2020). These factors underscore that changes in NTL radiance may not always represent true infrastructure improvement and highlight the importance of future validation with independent infrastructure datasets.

Third, further research could explore the interpretation of NTL trends that deviate from the five archetypes established in this study. Such deviations may result from the complexity and uncertainty of human activities, including factors such as civil unrest, natural disasters, and the ongoing COVID-19 pandemic. By fostering interdisciplinary collaboration between remote sensing communities and other disciplines — such as public health, demography, anthropology, and political science — we can further refine our understanding of the complex and multi-dimensional dynamics of urbanization in the Mediterranean region and beyond.

## 5. Conclusions

There is an urgent need to better understand the where, how, and when of multi-dimensional urbanization as a step toward anticipating risks to human and natural systems. While daytime and nighttime satellite images each have the capability to depict the status of urbanization, their individual use limits the range of dimensions that can be revealed. In this study, we examine the mismatches between nighttime light growth and urban expansion detected form daytime imagery using a confusion matrix. Our assessment shows 80% of urbanization-associated pixels exhibit either urban land expansion or lighting growth, but not both simultaneously. Commission error was higher than 50%, indicating most existing urban areas exhibit lighting growth, such as densification and road improvements. Conversely, 40% of urban expansion occurred without concurrent lighting growth, particularly in Western Europe and North Africa, which may indicate a lack of public infrastructure or energy conservation culture. In the future, more detailed analyses of NTL trends, combined with local knowledge and interdisciplinary collaboration, hold promise for providing a comprehensive understanding of urbanization processes.

## Supplementary Material

Supplementary material

## Figures and Tables

**Table 1:** Confusion matrix comparing predictions from nighttime satellite images (NTL) with reference data from daytime satellite images (WSF).

		WSF (Reference)	
	
		P: Urban expansion	N: No urban expansion

NTL	**P: Lighting growth**	TP: Urban expansion with lighting growth	FP: Lighting growth without urban expansion
	**N: No lighting growth**	FN: Urban expansion without lighting growth	TN: No urban expansion and no lighting growth

**Table 2:** Commission and omission errors in different regions, estimated when the WSF change threshold is set at 5%.

Region	Commission error (Lighting growth in non-expansion area, % of non-expansion area)	Omission error (Lacking lighting growth in expansion area, % of expansion area)

Eastern Europe	53.2%	42.5%
North Africa	51.7%	46.5%
West Asia	73.5%	20.3%
Western Europe	44.0%	52.2%

**All regions**	50.7%	43.4%

**Table 3:** Agreement and disagreement between daytime and nighttime signals across four regions around the Mediterranean Sea (unit: km^2^ (%)). The threshold for urban expansion is defined as a 5% change in the World Settlement Footprint for each 500m*500 m grid based on an elbow method.

Region	Urban expansion with lighting growth	No urban expansion or lighting growth	Urban expansion without lighting growth	Non-urban expansion with lighting growth	Total area

Eastern Europe	2771 (10%)	10,651 (39%)	2048 (7%)	12,125 (44%)	27,594
North Africa	13,831 (19%)	22,432 (31%)	12,042 (17%)	23,971 (33%)	72,275
West Asia	7449 (17%)	8986 (21%)	1895 (4%)	24,967 (58%)	43,297
Western Europe	7554 (5%)	72,639 (50%)	8234 (6%)	57,056 (39%)	145,482

**All regions**	31,605 (11%)	114,708 (40%)	24,219 (8%)	118,119 (41%)	288,651

## Data Availability

The datasets used in this study are publicly available. NASA Black Marble Nighttime Light data can be accessed at https://blackmarble.gsfc.nasa.gov/, and the World Settlement Footprint is available at https://geoservice.dlr.de/. The code used in this study is available at https://doi.org/10.5281/zenodo.18160091.

## Appendix A. Supplementary data

Supplementary material related to this article can be found online at https://doi.org/10.1016/j.jag.2026.105087.
