# Supplementary material for: Detecting gaps between urban expansion and lighting infrastructure growth using daytime and nighttime satellite imagery
Source: Int J Appl Earth Obs Geoinf. Author manuscript; Available in PMC 2026 Mar 25. (PMC13012652; doi:10.1016/j.jag.2026.105087)
Supplement: Supplementary material [file NIHMS2135680-supplement-Supplementary_material.docx]

Detecting Gaps Between Urban Land and Lighting Infrastructure Using Daytime and Nighttime Satellite Imagery
Tzu-Hsin Karen Chen, Wei Chen, Eleanor C. Stokes, and Yuyu Zhou

**Supplementary materials**

**K-shape clustering results**

**
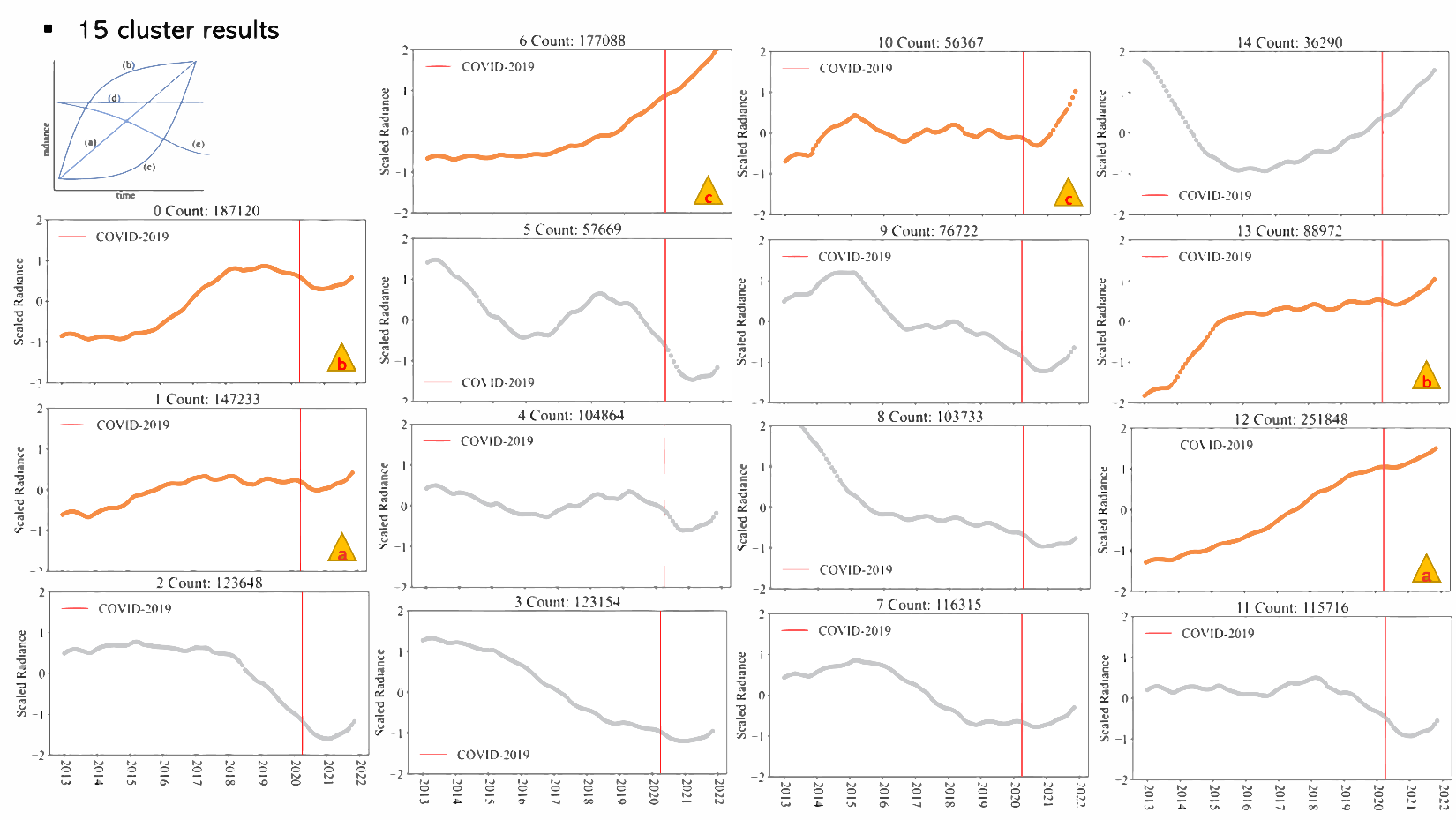
**

Fig. S1. Fifteen NTL time-series resulted from the K-shape clustering algorithm, which is grouped into either lighting infrastructure growth (orange) and not (gray).
